# Clinical efficacy and safety of faecal microbiota transplantation in the treatment of irritable bowel syndrome: a systematic review, meta-analysis and trial sequential analysis

**DOI:** 10.1186/s40001-024-02046-5

**Published:** 2024-09-18

**Authors:** Shao-Wei Lo, Tsung-Hsuan Hung, Yen-Tsen Lin, Chun-Shen Lee, Chiung-Yu Chen, Ching-Ju Fang, Pei-Chun Lai

**Affiliations:** 1 Education Centre, College of Medicine, National Cheng Kung University Hospital, National Cheng Kung University, No.138, Sheng Li Road, Tainan, 704 Taiwan; 2 Department of Internal Medicine, College of Medicine, National Cheng Kung University Hospital, National Cheng Kung University, Tainan, Taiwan; 3 Medical Library, National Cheng Kung University, Tainan, Taiwan; 4 Department of Secretariat, College of Medicine, National Cheng Kung University Hospital, National Cheng Kung University, Tainan, Taiwan; 5 Department of Paediatrics, College of Medicine, National Cheng Kung University Hospital, National Cheng Kung University, Tainan, Taiwan

**Keywords:** Irritable bowel syndrome, Faecal microbiota transplantation, Systemic review, Meta-analysis, Randomised controlled trial

## Abstract

**Background:**

The aim of this study is to evaluate the efficacy and safety of faecal microbiota transplantation (FMT) for the treatment of irritable bowel syndrome (IBS).

**Methods:**

We searched four databases for randomised controlled trials (RCTs) that compared FMT with a control intervention in patients with IBS. The revised Cochrane risk-of-bias (RoB) tool was chosen for appraisal. Meta-analysis with trial sequential analysis (TSA) was conducted. Grading of Recommendations Assessment Development and Evaluation (GRADE) methodology was used to assess the certainty of evidence (CoE).

**Results:**

We included 12 RCTs with a total of 615 participants. Meta-analyses showed no significant difference between the FMT and control groups in terms of clinical responses (relative risk [RR] = 1.44, 95% confidence interval [CI] 0.88–2.33) and changes in IBS Severity Scoring System (IBS-SSS) scores (standardised mean difference [SMD] =  − 0.31, 95% CI  − 0.72 to 0.09) and IBS Quality of Life (IBS-QOL) scores (SMD = 0.30, 95% CI  − 0.09 to 0.69). Subgroup analysis revealed that in studies with low RoB and using endoscopy, nasojejunal tube and rectal enema delivery, FMT led to a significant improvement in clinical responses and changes in IBS-SSS and IBS-QOL scores. TSA suggested that the current evidence is inconclusive and that the CoE is very low.

**Conclusion:**

This study suggests that patients with IBS may benefit from FMT especially when it is administered via endoscopy, nasojejunal tube or rectal enema. However, the certainty of evidence is very low. Further research is needed to confirm the efficacy and safety of FMT for IBS treatment.

*Trial Registration*: PROSPERO registration number CRD42020211002.

**Supplementary Information:**

The online version contains supplementary material available at 10.1186/s40001-024-02046-5.

## Introduction

Irritable bowel syndrome (IBS) is a functional gastrointestinal disorder characterised by abdominal pain and altered bowel habits, leading to considerable discomfort and impairing quality of life [1]. It affects a large proportion of the population, with prevalence rates ranging from 7 to 21% worldwide [2]. Despite its high prevalence, its underlying mechanisms are not yet fully understood, making developing effective treatments difficult [3].

Faecal microbiota transplantation (FMT) is a therapeutic intervention that involves the transfer of faecal microbiota from a healthy donor to a recipient with dysbiotic gut microbiota [4]. FMT is thought to work by restoring gut microbial diversity and function, which leads to an improvement in gastrointestinal and nongastrointestinal symptoms [5]. It has been used to treat a variety of disorders, including recurrent *Clostridioides difficile* infection (CDI), IBS, Parkinson’s disease and various inflammatory disorders [6]. In 2022, the FDA approved the first FMT therapy to prevent recurrent CDI in adults who previously completed antibiotic treatment [7]. This event represents a major milestone of FMT therapy.

Recent studies have suggested that alterations in gut microbiota may play a key role in the development and exacerbation of IBS symptoms [8]. The gut microbiota is a complex ecosystem of microorganisms that live in the gastrointestinal tract and has been shown to play a crucial role in maintaining gut homeostasis and overall health [9]. Dysbiosis or an imbalance in the gut microbiota has been associated with a variety of gastrointestinal disorders, including IBS [10]. This association has led to increasing interest in FMT as a potential therapy for IBS.

Although previous randomised controlled trials (RCTs) have evaluated the efficacy of FMT in IBS treatment, their results were inconsistent and limited by their small sample sizes [11–22]. Prior systematic reviews with meta-analyses of RCTs also have some limitations. Firstly, continuous outcomes, such as the IBS Severity Scoring System (IBS-SSS) and IBS Quality of Life (IBS-QOL), were pooled on the basis of their scores at the follow-up endpoint instead of the change in their scores from baseline [23, 24]. Furthermore, despite the existence of two different scoring systems for IBS-QOL in enrolled RCTs, the original scores, rather than the standardised mean difference (SMD), from each RCT were combined [24–26]. Last but not least, several new RCTs and full-text articles were not enrolled in previous meta-analyses [18–20]. We conducted an updated meta-analysis to provide a comprehensive and precise analysis of the available evidence on the efficacy and safety of FMT in treating IBS to address the above limitations. Our study also utilised trial sequential analysis (TSA) to provide a cautious evaluation of the data through repetitive and cumulative testing.

## Methods

### Literature searches and data sources

This systematic review and meta-analysis was conducted in accordance with the guidelines of the Cochrane Handbook of systematic reviews and followed the Preferred Reporting Items for Systematic Reviews and Meta-Analyses (PRISMA) statement [27]. We comprehensively searched online literature by using Embase, MEDLINE, Cochrane CENTRAL, CINAHL and Scopus. All the searches were completed on June 20, 2024. In addition, we searched clinicaltrials.gov manually for potential unpublished trials. The database search terms consisted of ‘irritable colon’ OR ‘irritable bowel syndrome’ AND ‘faecal microbiota transplantation’. We limited the results to ‘randomised controlled trials’ and ‘human’. The detailed search methods are available in the Supplementary Materials (supplementary Table 1). No language restrictions were imposed. We also manually searched the references of the included studies for additional relevant citations. Any further information required from the original author was requested through written correspondence (e.g. emailing the corresponding or first author).

### Study selection

Two authors (Shao-Wei, Lo and Tsung-Hsuan, Hung) independently screened the titles and abstracts then reviewed full texts and assessed the relevant studies for compliance with inclusion criteria. Any disagreement was resolved by discussion.

The inclusion criteria are as follows: (1) RCTs enrolling patients diagnosed with IBS by a clinician or on the basis of specific criteria, such as Rome Criteria or Manning. (2) Intervention with FMT at any dosage and through any route of administration and control group treated with a placebo or autologous transfer. (3) Primary outcomes included clinical response and changes in the severity of IBS symptoms, including IBS-SSS and IBS-QOL scores, and secondary outcomes included the safety and side effects of the intervention. The clinical response was the proportion of patients with clinical responses to the total examined patients and was defined as decreases of at least > 50 in IBS-SSS or > 30% in GSRS-IBS or adequate relief of global symptoms after 12 weeks.

The study design and protocol for this research were registered with PROSPERO. Approval was granted by its editorial team under registration number CRD42020211002.

### Data extraction and quality assessment

Two authors (Yen-Tsen Lin and Tsung-Hsuan Hung) independently extracted the data, including publication year, origin country, study design, sample size, patients’ baseline characteristics, IBS diagnosis criteria and subtype, intervention type, primary and secondary outcomes, adverse events, follow-up information and exclusion and inclusion criteria from the included studies. Two authors (Shao-Wei Lo and Chun-Shen Lee) independently assessed quality by using the revised Cochrane Risk-of-Bias tool for Randomised Trials (RoB 2) [28]. The confidence levels of the outcome effect estimates were evaluated by grading the quality of evidence as low risk, some concern for risk or high risk. Any disagreement was resolved by discussion.

### Statistical analysis

We used random-effects models to analyse pooled effect sizes and 95% confidence intervals (CIs) for all outcomes. For binary data, such as clinical response and adverse events, the meta-analysis used relative risk (RR). We analysed continuous data by using the standard mean difference (SMD) for the changes in IBS-SSS and IBS-QOL due to discrepancies amongst scales in the included studies. P-value < 0.05 was considered statistically significant. Heterogeneity was evaluated by using the I^2^ statistic [29].

TSA is a recently described cumulative frequentist meta-analysis method used to weigh type I and II errors and to provide information on the precision and uncertainty of the meta-analysis results. TSA also provides monitoring boundaries or futility boundaries to providing information on whether ongoing trials are necessary [30]. TSA version 0.9.5.10 beta was used in this study, and the details of model setting were mentioned in a previous report [31]. In summary, we analysed the data using a random-effects model via the Biggerstaff–Tweedie method, with a 5% type I error rate, 80% statistical power, and an improvement with a relative risk of 50%. The TSA result was presented as MD and α-spending adjusted CIs. We performed subgroup analysis on the basis of the following variables to explore possible causes leading to the heterogeneity of treatment effects: (1) the route of FMT delivery; (2) single or mixed donor samples; (3) fresh or frozen donor stool and (4) risk of bias (RoB) in the included studies. The differences in treatment effect were tested between subgroups, and *P* < 0.1 indicated a potential subgroup effect [27]. We detected publication bias with Egger’s test and funnel plots where more than 10 studies were present [32]. Data analysis and RoB plots were completed with Review Manager version 5.4 (Copenhagen: The Nordic Cochrane Centre, The Cochrane Collaboration, 2020).

### Grading of the certainty of evidence

The certainty of evidence is the extent to which we can be confident that what the research tells us about a particular treatment effect is likely to be accurate. The levels of evidence of all outcomes were assessed on the basis of the Grading of Recommendations Assessment Development and Evaluation (GRADE) methodology [33]. The overall certainty of evidence (CoE) was evaluated in accordance with the GRADE handbook by downgrading it to five domains [33]. The CoE was judged as high, moderate, low or very low and was constructed by using the online GRADE Profiler (available from http://www.gradepro.org).

## Results

### Search results and study characteristics

**Fig. 1:**
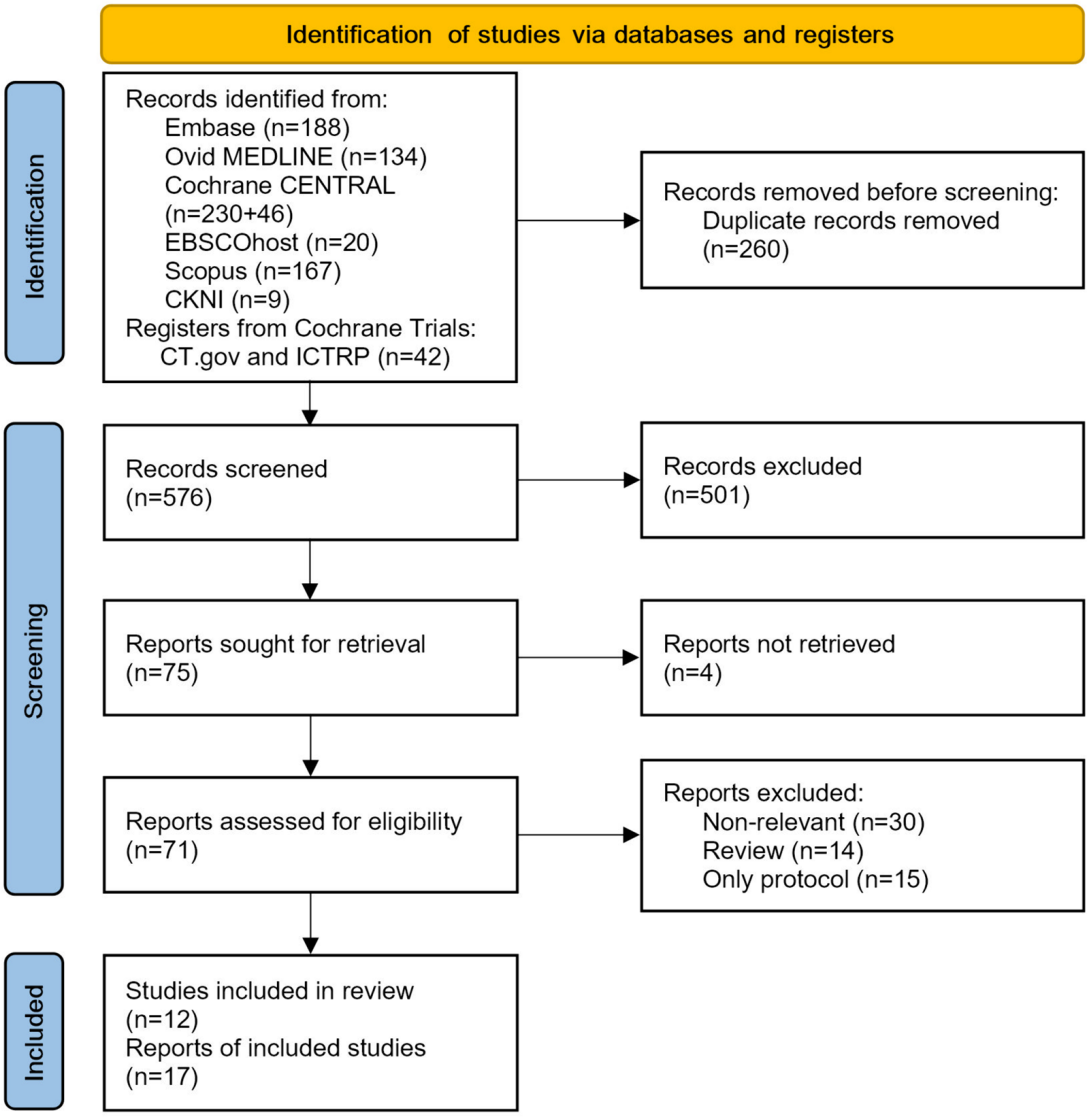
PRISMA 2020 flow diagram of study selection

Study selection is illustrated in Fig. 1. The initial literature search identified 639 potentially eligible articles. A total of 576 unique, relevant studies were retrieved after duplicate removal. After the titles and abstracts of the articles were screened, 71 eligible studies were retrieved and then subjected to full-text assessment. A total of 59 studies were excluded for various reasons, and 12 randomised trials were ultimately included in data extraction [11–22] (Fig. 1).

The included trials were published between 2018 and 2023. When all the studies were combined, the total numbers of patients in the FMT and control groups were 356 and 259, respectively. A summary of the included studies and the baseline characteristics of the enrolled participants are presented in Table 1.

**Table 1:** The characteristics of included studies

Author, year (country)	Sample size (female)	IBS-criteria and subtypes	FMT intervention	Placebo intervention	Primary outcomes	Secondary outcomes
Halkjaer et al., 2018 (Denmark)	52 (35)	Rome III, IBS-SSS ≥ 175, 33.3% IBS‐C, 29.4% IBS‐D, 37.3% IBS‐M	25 FMT oral capsules consisting of 50 g frozen donor stool daily × 12d Mixed samples of 4 donors	26 placebo oral capsules daily × 12d	Decrease in IBS-SSS ≥ 50 points at 3 months	Change in IBS-QOL, microbiota profile, adverse event
Johnsen et al., 2018 (Norway)	83 (55)	Rome III, IBS-SSS ≥ 175, 53.0% IBS‐D, 47.0% IBS‐M	Single FMT consisting of 50–80 g both fresh and frozen (1:1) donor stool to the cecum via colonoscopy Mixed samples of 2 donors	50‐80 g autologous stool via colonoscopy	Decrease in IBS-SSS ≥ 75 points at 3 months	Change in IBS-QOL, adverse event
Holster et al., 2019 (Sweden)	16 (8)	Rome III, 25.0% IBS‐C, 56.2% IBS‐D, 18.8% IBS‐M	Single FMT consisting of 30 g frozen donor stool to the cecum via colonoscopy Single sample of either of the 2 donors	30 g autologous stool via colonoscopy	Decrease in GSRS-IBS ≥ 30%	Change in IBS-QOL, IBS-SSS, microbiota profile, anxiety, depression, adverse event
Aroniadis et al., 2019 (USA)	48 (18)	Rome III, IBS-SSS ≥ 175, 100% IBS-D	25 FMT oral capsules consisting of 28 g frozen donor stool daily × 3d Single sample of either of the 4 donors	25 placebo oral capsules daily × 3d	Decrease in IBS-SSS ≥ 50 points at 12 weeks	Change in IBS-QOL, depression, anxiety, stool form, microbiota profile, adverse event
El-Salhy et al., 2019 (Norway)	164 (133)	Rome IV, IBS-SSS ≥ 175, 38.4% IBS-D, 37.8% IBS-C, 23.8% IBS-M	Single FMT consisting of 30 g or 60 g frozen donor stool to the distal duodenum via gastroscopy A single donor	Single autologous stool via gastroscopy	Decrease in IBS-SSS ≥ 50 points at 3 months	Change in IBS-QOL, dysbiosis index, microbiota profile
Lahtinen et al., 2020 (Finland)	49 (29)	Rome III, 51.0% IBS‐D, 6.1% IBS‐C, 14.3% IBS‐M, 28.6% IBS-U	Single FMT consisting of 30 g frozen donor stool to the cecum via colonoscopy A single donor	Single 30 g autologous stool via colonoscopy	Decrease in IBS-SSS ≥ 50 points at 12 weeks	Change in IBS-QOL, depression, anxiety, stool consistency, microbiota profile
Guo et al., 2021 (China)	18 (8)	100% IBS-D comorbid with HAM-A ≥ 14 and HAM-D ≥ 8	30 FMT oral enteric capsules, per 2 days, for 3 times	30 empty oral capsules	Decrease in IBS-SSS ≥ 50 points at 12 weeks	Change in IBS-QOL, depression, anxiety, microbiota profile
Holvoet et al., 2021 (Belgium)	62 (38)	Rome III, refractory IBS with severe bloating, IBS-D, IBS-M	Single FMT consisting of fresh donor stool via naso-jejunal tube Single sample of either of the 2 donors	Single autologous stool via naso-jejunal tube	Adequate relief of overall symptoms at 12 weeks	Change in IBS-QOL, IBS symptom, stool consistency, microbiota profile, adverse event
Aumpan et al., 2022 (Thailand)	20 (N/A)	Rome IV	Single FMT consisting of 50 g donor stool via rectal enema	Single 50 g autologous stool via rectal enema	Decrease in IBS-SSS ≥ 50 points	change in IBS-QOL, abdominal pain, abdominal distention
Mazzawi et al., 2022 (Norway)	26 (N/A)	Rome III, IBS-SSS ≥ 175, 100% IBS-D	Single FMT consisting of 30 g fresh donor stool via gastroscopy A Single donor from healthy family members (first-grade relatives)	Single 30 g autologous stool via gastroscopy	Decrease in IBS-SSS ≥ 50 points (Not reported)	Change in IBS-SSS, stool consistency, anxiety, depression, microbiota profile
Singh et al., 2022 (USA)	23 (11)	Rome III, IBS-SSS > 150, 100% IBS-D	19 FMT oral capsules, each pill consisting of 0.75 g of frozen donor stool A single donor	19 placebo oral capsules	Decrease in IBS-SSS ≥ 50 points	Change in IBS-SSS, IBS-QOL, microbiota profile, adverse event
Yau et al., 2023 (China)	56 (26)	Rome III	100 mL of FMT with 50 g donor stool was infused via upper endoscopy into distal duodenum at baseline and week 4 9 patients received stool from two donors, and the remaining 19 patients received stool from one donor	100 mL of normal saline infused via upper endoscopy into the distal duodenum under conscious sedation at baseline and week 4	Decrease in IBS-SSS ≥ 50 points	Change in IBS-SSS, relief of general IBS symptoms, quality of life, faecal microbiome metagenomic profiling

### RoB

The RoB assessment domains and authors’ judgments with justifications based on the RoB 2.0 tool were summarised (Supplementary Fig. 1). Five studies were of some concern for allocation bias because no information was available about concealment, [11, 16–18, 20]Additionally, one study [22] exhibited imbalanced baseline characteristics between the two groups post-randomisation, including age and baseline IBS severity, which could potentially confound the true effect. Only one study was rated with high RoB for the domain of performance bias because information about the blinding of the participants and personnel was unavailable and the authors did not appropriately analyse the effect of adherence. [18] Four studies were of some concern for RoB in the attrition bias domain due to their relatively high and unequal dropout rates without available explanations [12, 14, 19, 20]. Two studies were of some concern for detection bias because no information was available about the assessors being blinded. [16, 18] Overall, two of the included studies had low RoB [13, 15]; six were of some concern for RoB [12, 14, 17, 19, 20, 22]; and three had high RoB [11, 16, 18]. The RoB of the study of Aumpan et al., 2022 could not be assessed because only its abstract was published.

### Outcome

#### Comparison of the clinical response of the FMT group with that of the control group after 12 weeks

**Fig. 2:**
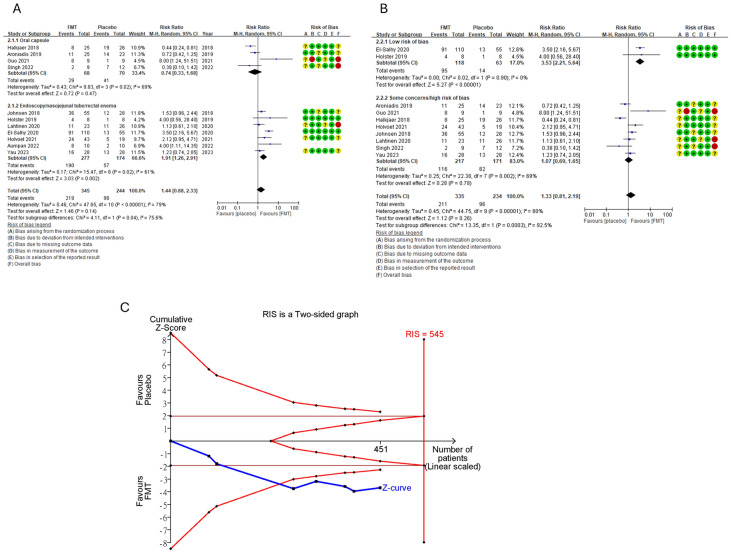
Forest plot of the clinical responses of patients with IBS to FMT or the placebo. **a** Subgroup analysis based on the route of FMT delivery. **b** Subgroup analysis based on the RoB of a study. **c** TSA of the FMT treatment effect on patients with IBS

The pooled effect showed no significant difference between the FMT and control groups in clinical response rate with high heterogeneity (RR = 1.44, 95% CI 0.88–2.33, *I*^2^ = 79%) (Fig. 2a). We further conducted subgroup analyses in accordance with the route of FMT delivery (Fig. 2a), the RoB of each study (Fig. 2b), the use of a single or mixed donor sample (Supplementary Fig. 2a) and fresh or frozen donor stool (Supplementary Fig. 2b) to explore potential heterogeneity. The clinical response of the group that received FMT via endoscopy (colonoscopy [12, 13, 16] and gastroscopy [15, 22]), nasojejunal tube [17] and rectal enema [21] was superior to that of the control group with moderate heterogeneity (RR = 1.91, 95% CI 1.26–2.91, *I*^2^ = 61%) (Fig. 2a). The pooled effect showed no statistically significant difference between FMT and the control administered via oral capsule [11, 14, 18, 20] (RR = 0.73, 95% CI 0.32–1.68, I^2^ = 70%) (Fig. 2a). Pooled data from RCTs with a low RoB revealed that the FMT group was superior to the control groups with low heterogeneity (RR = 3.53, 95% CI 2.21–5.64, *I*^2^ = 0%) (Fig. 2b) [13, 15]. By contrast, the pooled effect showed no statistically significant difference between the FMT and control groups in studies with some concerns [11, 12, 14, 17, 22] or high RoB [16, 18, 20] (RR = 1.07, 95% CI 0.69–1.65, *I*^2^ = 69%) (Fig. 2b).

Because the subgroup analysis of FMT via direct delivery methods (including endoscopy, nasojejunal tube and rectal enema) showed more promising results, we performed further post hoc analysis by TSA in this subgroup. The Z-curves crossed the O'Brien–Fleming α-spending monitoring boundaries, indicating a potentially significant effect. Nevertheless, the sample size included in this subgroup analysis did not exceed the required information size, limiting the findings' strength (Fig. 2c).

#### Change in IBS‐SSS scores from the baseline after 8–12 weeks

**Fig. 3:**
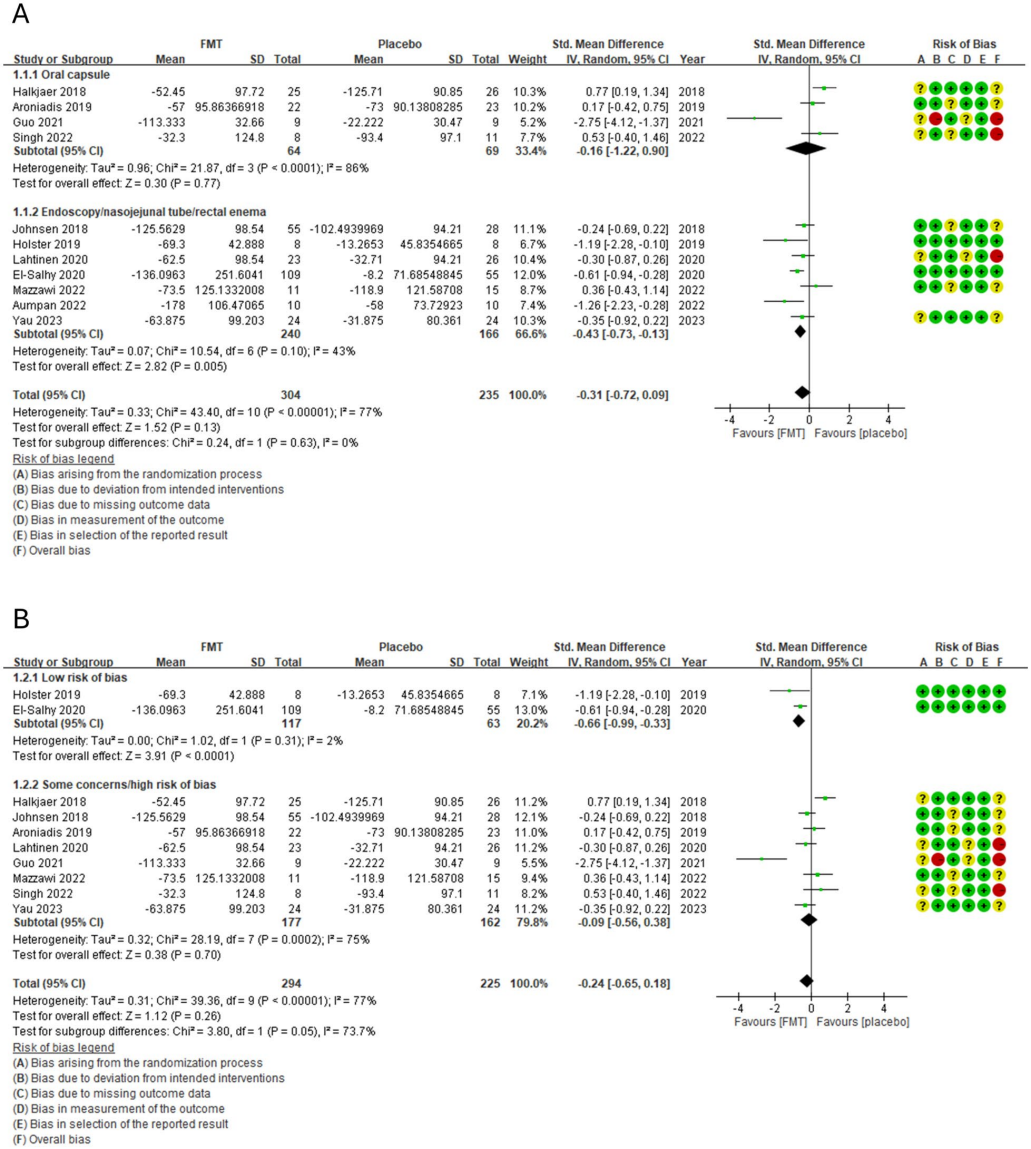
Forest plot of the change in the IBS-SSS of patients with IBS in response to FMT or the placebo. **a** Subgroup analysis based on the route of FMT delivery. **b** Subgroup analysis based on the RoB in a study

The overall pooled estimates revealed no statistically significant difference in IBS-SSS scores after 8–12 weeks between the FMT and control groups with high heterogeneity (SMD =  − 0.31, 95% CI  − 0.72 to 0.09, I^2^ = 77%) (Fig. 3a). Subgroup analyses showed that FMT delivered via endoscopy [12, 13, 15, 16, 19, 22], nasojejunal tube and rectal enema had significantly reduced IBS-SSS scores after 8–12 weeks compared with the control with low heterogeneity (SMD =  − 0.43, 95% CI  − 0.73 to  − 0.13, *I*^2^ = 43%) (Fig. 3a). No statistically significant difference was found between the FMT and control groups treated with oral capsules [11, 14, 18, 20] (SMD =  − 0.16, 95% CI  − 1.22 to 0.90, *I*^2^ = 86%) (Fig. 3a). Pooled data from RCTs with a low RoB [13, 15] indicated that FMT was superior to the control with low heterogeneity (SMD =  − 0.66, 95% CI  − 0.99 to  − 0.33, *I*^2^ = 2%) (Fig. 3b).

#### Change in IBS‐QOL scores from the baseline after 8–12 weeks

**Fig. 4:**
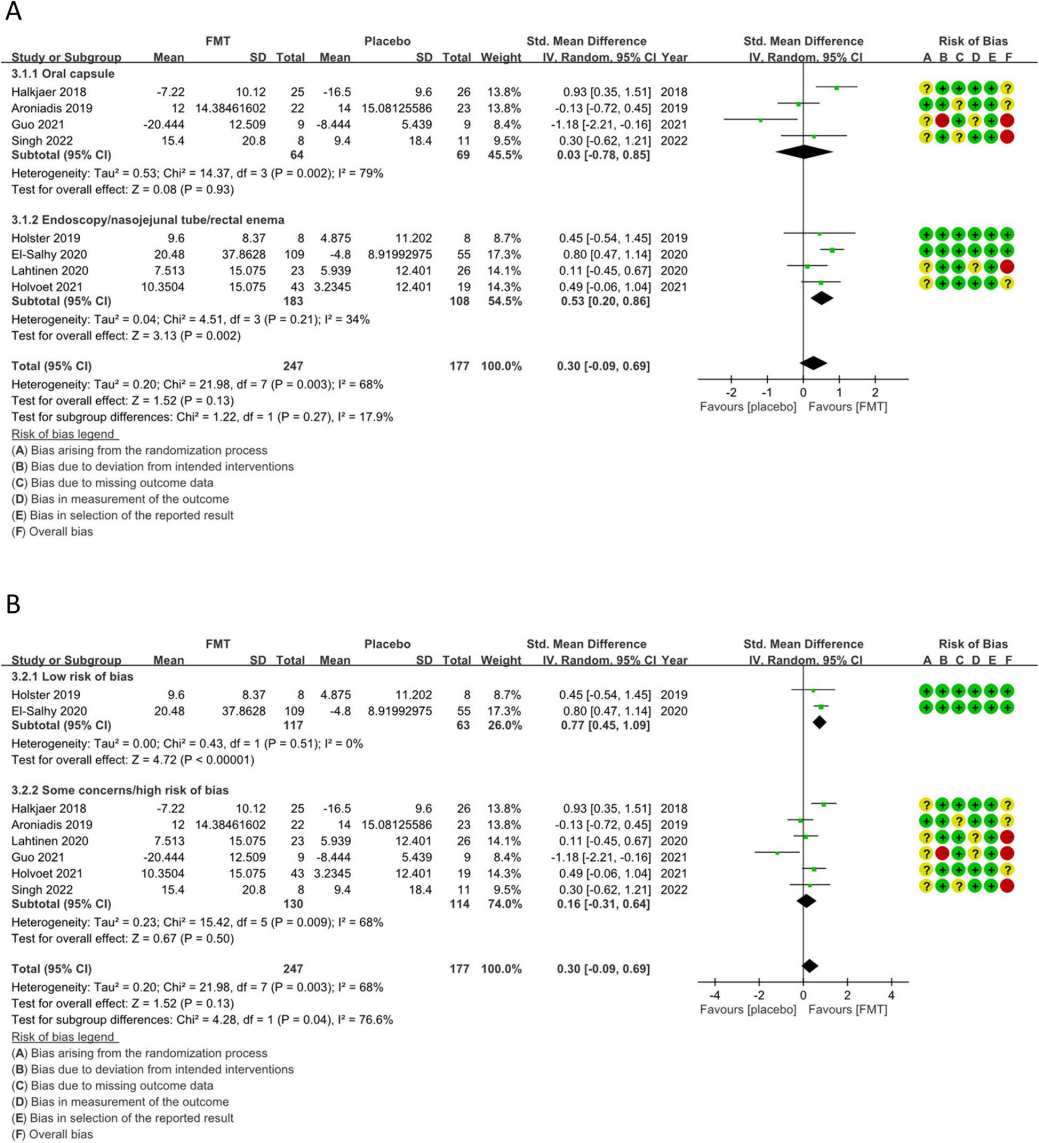
Forest plot of the change in the IBS-QOL of patients with IBS in response to FMT or the placebo. **a** Subgroup analysis based on the route of FMT delivery. **b** Subgroup analysis based on the RoB of a study

Pooled estimates showed no statistically significant difference in IBS-QOL scores after 8–12 weeks between the FMT and control groups (SMD = 0.30, 95% CI  − 0.09 to 0.69, *I*^2^ = 68%) (Fig. 4a). Subgroup analyses demonstrated that FMT delivered via endoscopy [13, 15, 16], nasojejunal tube [17] and rectal enema significantly improved IBS-QOL scores after 8–12 weeks compared with the control (SMD = 0.53, 95% CI 0.20–0.86, *I*^2^ = 34%) (Fig. 4a). However, the pooled effect showed no statistically significant difference between the FMT and control delivered via oral capsule [11, 14, 18, 20] (SMD = 0.03, 95% CI  − 0.78 to 0.85, *I*^2^ = 79%) (Fig. 4a). Pooled data from RCTs with a low RoB [13, 15] showed that FMT was superior to the control with low heterogeneity (SMD = 0.77, 95% CI 0.45–1.09, *I*^2^ = 0%) (Fig. 4b).

#### Adverse events

**Fig. 5:**
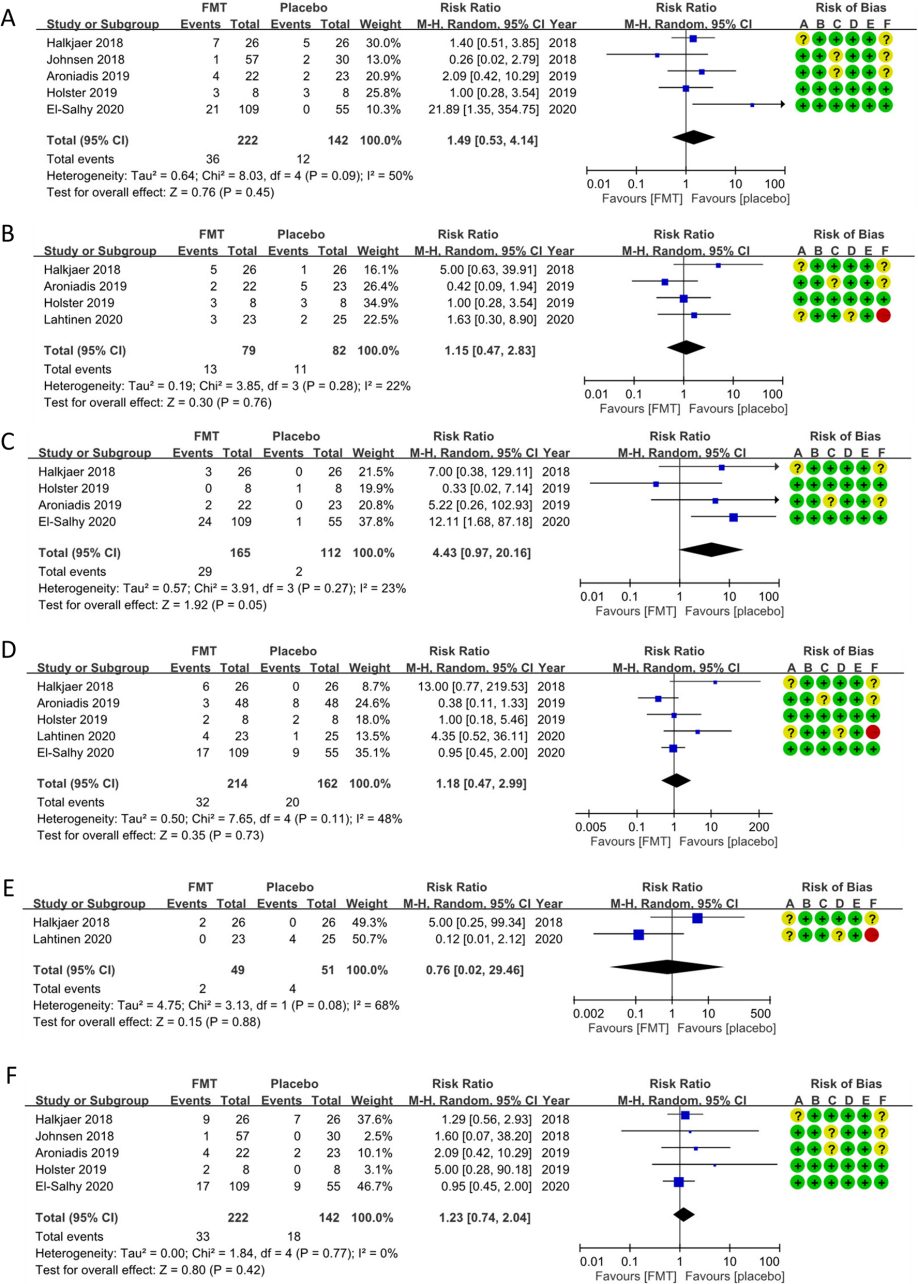
Forest plot of the adverse events in patients with IBS in response to FMT or the placebo: **a** abdominal pain, **b** bloating/flatulence, **c** constipation, **d** diarrhea, **e** fever, **f** nausea

Pooled analysis revealed no significant difference between the FMT and control groups in adverse events, including nausea, abdominal pain/cramping/tenderness, diarrhoea, constipation, bloating/flatulence and fever (Fig. 5).

#### GRADE assessment

The certainty of evidence in consideration of the outcomes with clinical response and changes in IBS-SSS and IBS-QoL scores were all judged as ‘very low’ in accordance with GRADE criteria. We downgraded the CoE in the domain of risk of bias because more than half of the included studies were judged as having some concern to high RoB. We also downgraded in the domain of inconsistency due to high heterogeneity, and in the domain of imprecision due to wide confidence intervals. (Supplementary Table 2). Indirectness and publication bias were not considered with the Egger’s regression test (*P* = 0.72725) and the funnel plot showed no evidence of publication bias (Supplementary Fig. 3).

## Discussion

IBS is a prevalent gastrointestinal disorder that significantly impacts patients' quality of life and imposes substantial economic burdens globally [34, 35]. Recent research has delved into the potential of microbiota-targeting treatments for IBS sufferers [36]. The rationale behind these interventions hinges on the direct influence of microbiota on the gut’s mucosal environment and their regulatory impact on the gut–brain communication pathway [36], aiming to preserve gut mucosal integrity, alter gut microbiome composition and mitigate inflammatory cytokine release [37, 38]. However, the definitive effectiveness and safety of FMT in IBS patients remain inconclusive.

To address this gap, our study performed a comprehensive systematic review and meta-analysis to explore the efficacy and safety of FMT in patients with IBS. We included updated studies and eventually identified 12 RCTs. In addition, we combined various scoring systems in IBS-QOL by using SMD while considering the changes in IBS-SSS and IBS-QOL scores from the baseline. We further strengthened our analysis by applying the rigorous TSA method to test the robustness of our findings. As a result, our study provides a highly reliable and insightful perspective on the effectiveness of FMT in IBS treatment.

Consistent with the meta-analyses published recently in 2022 and 2023, our study revealed no significant differences between the FMT and control groups in terms of clinical responses after 12 weeks [23, 24, 26, 39]. A similar result was shown in terms of the changes in IBS-SSS and IBS-QOL after 8–12 weeks. We also observed high heterogeneity amongst studies, suggesting that caution is needed in interpreting results. No serious adverse events were related to FMT in IBS. The GRADE assessments indicated that the CoE for all clinical outcomes in our study was very low.

Various delivery routes are currently utilised for FMT. They include oesophagogastroduodenoscopy; nasogastric, nasojejunal or nasoduodenal tube; colonoscopy; rectal enema and oral capsule delivery. Our subgroup analyses revealed that the route of FMT delivery significantly influenced its efficacy. FMT delivered via routes with more direct delivery to the gut, such as endoscopy, nasojejunal tube, or rectal enema, significantly improved all three outcomes, including clinical response, change in IBS-SSS and change in IBS-QOL compared to control. In contrast, oral capsule FMT did not demonstrate any benefits, suggesting that direct delivery to the target site may be more effective. Several biological and physiological factors may explain the superiority of direct delivery routes. Firstly, the live bacterial counts of FMT capsules range widely from 100 million bacteria per capsule to 100 billion bacteria per capsule and decline rapidly over time, reaching only 10% of their initial values after 24 hours [40]. This rapid decline in bacterial viability can significantly impact the effectiveness of the treatment. Secondly, the capsules expose bacteria to harsh gastric conditions, reducing bacterial viability and colonisation potential in the gut [41, 42]. Direct delivery methods like endoscopic, nasojejunal and rectal enema bypass the stomach, allowing for better survival and engraftment of the transplanted microbiota. [43] Thirdly, the gastrointestinal tract, particularly the lower intestines, is an anaerobic environment. Many of the beneficial gut microorganisms, such as obligate anaerobes, are highly sensitive to oxygen exposure [44]. Oral capsules may expose these anaerobic microorganisms to oxygen during transit through the upper gastrointestinal tract, potentially compromising their viability and functionality. Direct delivery minimises exposure to oxygen, maintaining the anaerobic environment necessary for these microorganisms to thrive. [45] Lastly, deviations from recommended protocols, such as using suboptimal doses or improper storage conditions, may have decreased the efficacy of oral capsule FMT in some studies. As noted previously by Rodrigues et al. [39] the recommended dose for a faecal transplant is 30 g. However, Aroniadis et al. administered less than the recommended dose. [14] In addition, Halkjaer et al. stored their final faecal suspensions at − 20 °C [11], whereas guidelines suggest storage at − 80 °C [46]. These deviations from the recommended protocol may have decreased the efficacy of oral capsule FMT, thereby diminishing the overall pooled effect of its efficacy.

Another emerging way to deliver FMT is colonic transendoscopic enteral tubing (TET), which has shown potential in treating various gut disorders, including IBS [47]. This procedure involves inserting a long, soft tube through the rectum into the colon using a colonoscope to infuse the faecal suspension directly into the colonic region. This tube allows for targeted infusion of the faecal suspension throughout the colonic region [47]. Compared to traditional colonoscopic delivery, TET is less invasive, better tolerated by patients and eliminates the need for full colon preparation [47]. Growing evidence suggests that administering FMT through a colonic TET could serve as a promising and more patient-friendly treatment strategy for patients with inflammatory bowel disease [47]. A recent prospective observational study by Zhang et al. demonstrated that washed microbiota transplantation delivered via mid-gut TET in 12 patients (16.4%) and colonic TET in 61 patients (83%) effectively improved both gastrointestinal and extraintestinal symptoms in individuals with IBS [48]. Despite these promising findings, further research through rigorous clinical trials specifically evaluating colonic TET-delivered FMT for IBS treatment is necessary.

The quality of the pooled studies could affect the reported effectiveness of FMT treatment. In our study, subgroup analysis based on overall RoB showed that in studies with a low RoB, the patients who received FMT had a significant improvement in all clinical outcomes, suggesting that methodological rigour is crucial in evaluating the true efficacy of FMT. Potential biases like inadequate allocation concealment, lack of blinding and high dropout rates may have obscured true effects in lower-quality studies. Further large-scale, high-quality RCTs are warranted to confirm the therapeutic role of FMT in the management of IBS.

Our study also analysed the effect of different faecal origins on FMT and its efficacy in patients with IBS. The results of subgroup analysis did not reveal a significant clinical response to FMT samples from single or mixed donors and in patients who received FMT using fresh, frozen or mixed stool samples. However, due to the limited sample size of our study, further research is needed to reach a conclusion on the preferable type of faeces.

The present study has several limitations. One major limitation is the heterogeneity of the enrolled participants, which persisted even after extensive subgroup analyses. This heterogeneity can be attributed to several factors. Firstly, the enrolled participants exhibited high heterogeneity, with variations in the diagnostic criteria employed IBS as well as the specific IBS subtypes represented. These RCTs included participants with different IBS subtypes (IBS-C, IBS-D, IBS-M, IBS-U) and most of the RCTs included a mixture of patients with differing IBS subtypes, which may respond differently to FMT due to varying underlying pathophysiologies. This variation makes it challenging to determine whether FMT efficacy differs among IBS subtypes. Future studies should focus on specific IBS subtypes to identify patient populations most likely to benefit from FMT.

Secondly, the inclusion criteria for symptom severity varied across studies. Some included participants with more severe IBS symptoms (e.g. IBS Symptom Severity Score (IBS-SSS) ≥ 175) [11, 12, 14, 15, 20], while others did not specify symptom severity criteria, potentially leading to differences in treatment response [13, 16, 18, 19, 21]. Furthermore, utilising different diagnostic criteria, Rome IV versus Rome III, leads to the inclusion of distinct patient populations with varying disease severities, as Rome IV criteria tend to identify individuals with a more severe clinical presentation of IBS [48, 49]. This discrepancy in the recruited cohorts based on diagnostic criteria introduces a fundamental difference in the study populations, complicating the interpretation and comparison of treatment outcomes across studies. Future studies should focus on implementing standardised symptom severity criteria and unified diagnostic standards (preferably Rome IV) across all trials. Studies are recommended to incorporate pre-planned subgroup and sensitivity analyses to evaluate the impact of different inclusion criteria on outcomes.

Thirdly, the FMT interventions differed in terms of their origin, dosage, therapy duration, frequency, comparators and study protocols, making it difficult to analyse and compare the results. The dosages of donor stool ranged from 25 capsules (50g) to a single dose of 30-80g, and the frequency of administration varied from a single dose to multiple doses over several days. The placebo interventions varied across studies, with some using autologous stool transplantation and others using inert capsules or solutions. These variations may differently impact the gut microbiota, placebo response and the relative efficacy of FMT. Additionally, several studies lacked clear reporting of the inclusion and exclusion criteria used for donor selection, which may limit the generalisability of the findings. To enhance consistency and reproducibility, future trials should adopt a comprehensive, standardised protocol that includes donor screening and selection, FMT dosage, frequency, duration and placebo interventions, all guided by the latest consensus statements on best practices for FMT [50–52].

The heterogeneity observed in our meta-analysis, arising from the diverse features discussed, underscores the complex and multifaceted nature of both IBS and FMT as a therapeutic intervention. Although this heterogeneity limits the robustness of our conclusions, it also offers valuable insights into the factors that may influence the efficacy of FMT in treating IBS.

Another limitation of our study is that the included RCTs primarily focused on gut-specific symptoms like abdominal pain, bloating and bowel habits (i.e. IBS-SSS) and their impacts on quality of life (i.e. IBS-QOL). However, it’s crucial to recognise that IBS is a multifaceted condition that affects more than just the gastrointestinal system, and these broader effects can significantly impair a patient’s quality of life [53]. Psychological distress, including anxiety and depression, is common among IBS patients, influencing various aspects like gut physiology and immune response through the gut–brain axis [54]. Unfortunately, most RCTs do not adequately capture these outcomes, leading to a high degree of variability among studies that do include them. Guo et al. focused on patients with IBS-D who also suffered from depression and anxiety [18]. Their findings revealed that FMT therapy reduced not only gastrointestinal symptoms but also anxiety and depression. [18] Conversely, studies by Aroniadis et al., Mazzawi et al., Holster et al. and Lahtinen et al. reported no significant effect of FMT on depression and anxiety [13, 14, 16, 19]. Notably, Mazzawi et al. and Lahtinen et al. did not provide baseline data on depression and anxiety [16, 19], and Holster et al. explicitly excluded patients with depression prior to intervention [13]. Factors like concurrent psychological disorders, diet variations, co-medications and follow-up care are often overlooked in current RCTs, potentially limiting the efficacy evaluation of FMT for IBS [55]. Future research should adopt a comprehensive approach, including standardised tools to assess not only gastrointestinal symptoms but also psychological health and other relevant outcomes.

Despite these limitations, our study provides valuable insights into the effectiveness of FMT for IBS treatment. While the overall pooled estimates did not show a significant benefit of FMT, the subgroup analyses suggest that FMT, particularly when delivered via endoscopy, nasojejunal tube, or rectal enema, and in well-designed studies, may be an effective treatment option for improving symptoms and quality of life in IBS patients. However, the certainty of evidence was rated as “very low” due to concerns about bias, heterogeneity and imprecision, indicating limited confidence in the effect estimates. The true effect may differ from the estimates presented in our meta-analysis. Although the TSA results for the most important outcome, clinical response, suggest that the current evidence is a true positive, the sample size remains insufficient to draw a definitive conclusion. This inadequacy in sample size leads to a downgrade in the GRADE assessment in the domain of imprecision. Further well-designed studies with more participants should strive to standardise study designs, donor screening, treatment protocols, outcome metrics and the stratification of participants by IBS subtype to enhance the consistency and applicability of FMT research in IBS. It is imperative to explore potential effect modifiers through pre-specified subgroup analyses and meta-regression, as well as to examine the long-term effects and safety of FMT, to effectively integrate these findings into clinical practice.

## Conclusion

This study revealed that while the overall pooled estimates did not show a significant benefit of FMT for IBS, subgroup analyses revealed that FMT delivered via routes with more direct delivery to the gut, such as endoscopy, nasojejunal tube, or rectal enema, and in well-designed studies, may be an effective treatment option for improving symptoms and quality of life in IBS patients. The overall certainty of evidence was very low and the TSA indicated that the current evidence is inconclusive. Therefore, larger well-designed randomised controlled trials with rigorous methodology are warranted. Future studies should aim to standardise protocols for donor screening, treatment regimens and outcome assessments to enhance the consistency and clinical applicability of findings.

## Supplementary Information

Supplementary material 1: Table 1. Search strategy

Supplementary material 2: Table 2. GRADE evidence profile: FMT for patients with IBS

Supplementary material 3: Figure 1. RoB summary

Supplementary material 4: Figure 2. Forest plot of the clinical responses of patients with IBS to FMT or the placeboSubgroup analysis based on FMT samples from single or mixed donor stoolSubgroup analysis based on FMT stool from a fresh, frozen or mixed sample

Supplementary material 5: Figure 3. Funnel plot with Egger’s test

## Data Availability

Most of the data generated or analysed during this study are included in this published article and its supplementary information flies. Other datasets associated with the current study are available from the corresponding author on reasonable request.
