# Supplementary material for: Clinical efficacy and safety of faecal microbiota transplantation in the treatment of irritable bowel syndrome: a systematic review, meta-analysis and trial sequential analysis
Source: Eur J Med Res. 2024 Sep 18;29:464. doi: 10.1186/s40001-024-02046-5 (PMC11409544; doi:10.1186/s40001-024-02046-5)
Supplement: Supplementary file 1 — Supplementary material 1: Table 1. Search strategy [file 40001_2024_2046_MOESM1_ESM.docx]

**Search Strategy**

| Database | # | Search syntax |
| --- | --- | --- |
| **Embase (Elsevier)** | 1 | (**(**(irritable or unstable or nervous or spasm* or spastic* or mucomembran* or mucous*) NEAR/4 (colon* or bowel* or colitis or colitides)**)** or "functional colon*" or colonospasm* or ibs):ti,ab,kw,de |
|  | 2 | " irritable colon"/exp |
|  | 3 | (**(**(feces or faeces or fecal or faecal or stool*) NEAR/4 (enema* or infusion* or transplant* or instill* or tranfus* or donor* or reconstitution* or therap*)**)** or **(**(matter* or microb* or bacteri* or microflora*) NEAR/4 (transplant* or transfer*)**) or** bacteriotherap* or FMT):ti,ab,kw,de |
|  | 4 | "fecal microbiota transplantation"/exp |
|  | 5 | (#1 OR #2) AND (#3 OR #4) AND [embase]/lim |
|  | 6 | #5 AND ("randomized controlled trial"/de or "controlled clinical study"/de or "randomization"/de or "intermethod comparison"/de or "double blind procedure"/de or "human experiment"/de OR (random* or placebo or "parallel group$" or crossover or "cross over" or assigned or allocated or volunteer or volunteers):ti,ab OR (open NEAR/1 label):ti,ab OR ((double or single or doubly or singly) NEAR/1 (blind or blinded or blindly)):ti,ab OR ((assign* or match or matched or allocation) NEAR/5 (alternate or group$ or intervention$ or patient$ or subject$ or participant$)):ti,ab OR (controlled NEAR/7 (study or design or trial)):ti,ab OR (compare or compared or comparison or trial):ti OR ((evaluated or evaluate or evaluating or assessed or assess) and (compare or compared or comparing or comparison)):ab) NOT (((random* NEAR/1 sampl* NEAR/7 ("cross section*" or questionnaire$ or survey* or database$)):ti,ab not ("comparative study"/de or "controlled study"/de or "randomi$ed controlled":ti,ab or "randomly assigned":ti,ab)) OR ("cross-sectional study"/de not ("randomized controlled trial"/de or "controlled clinical study"/de or "controlled study"/de or "randomi$ed controlled":ti,ab or "control group$":ti,ab)) OR ((((case NEAR/1 control*) and random*) not "randomi$ed controlled"):ti,ab) OR (("systematic review" not (trial or study)):ti) OR ((nonrandom* not random*):ti,ab) OR ("random field*":ti,ab) OR (("random cluster" NEAR/3 sampl*):ti,ab) OR ((review:ab and review/it) not trial:ti) OR ("we searched":ab and (review:ti or review/it)) OR ("update review":ab) OR ((databases NEAR/4 searched):ab) OR ((rat or rats or mouse or mice or swine or porcine or murine or sheep or lambs or pigs or piglets or rabbit or rabbits or cat or cats or dog or dogs or cattle or bovine or monkey or monkeys or trout or marmoset*):ti and "animal experiment"/de) OR ("animal experiment"/de not ("human experiment"/de or "human"/de))) |
|  | 7 |  |
|  | 8 |  |
| **MEDLINE (Ovid)** | 1 | (**(**(irritable or unstable or nervous or spasm* or spastic* or mucomembran* or mucous*) ADJ4 (colon* or bowel* or colitis or colitides)**)** or "functional colon*" or colonospasm* or ibs).mp |
|  | 2 | exp " **Irritable Bowel Syndrome**"/ |
|  | 3 | (**(**(feces or faeces or fecal or faecal or stool*) ADJ4 (enema* or infusion* or transplant* or instill* or tranfus* or donor* or reconstitution* or therap*)**)** or **(**(matter* or microb* or bacteri* or microflora*) ADJ4 (transplant* or transfer*)**) or** bacteriotherap* or FMT).mp |
|  | 4 | exp " Fecal Microbiota Transplantation"/ |
|  | 5 | (1 OR 2) AND (3 OR 4) |
|  | 6 | 5 AND (randomized controlled trial.pt. or controlled clinical trial.pt. or randomized.ab. or randomised.ab. or placebo.ab. or drug therapy.fs. or randomly.ab. or trial.ab. or groups.ab. not (exp animals/ not humans.sh.)) |
|  | 7 |  |
|  | 8 |  |
| **Cochrane CENTRAL** | 1 | (**(**(irritable or unstable or nervous or spasm* or spastic* or mucomembran* or mucous*) NEAR/3 (colon* or bowel* or colitis or colitides)**)** or "functional colon*" or colonospasm* or ibs):ti,ab,kw |
|  | 2 | [mh "**Irritable Bowel Syndrome**"] |
|  | 3 | (**(**(feces or faeces or fecal or faecal or stool*) NEAR/3 (enema* or infusion* or transplant* or instill* or tranfus* or donor* or reconstitution* or therap*)**)** or **(**(matter* or microb* or bacteri* or microflora*) NEAR/3 (transplant* or transfer*)**) or** bacteriotherap* or FMT):ti,ab,kw |
|  | 4 | [mh "Fecal Microbiota Transplantation"] |
|  | 5 | (#1 OR #2) AND (#3 OR #4) |
|  | 6 | #5 (Limits: in Trials) |
|  | 7 |  |
|  | 8 |  |
| **CINAHL (EBSCOhost)** | 1 | **(**(irritable or unstable or nervous or spasm* or spastic* or mucomembran* or mucous*) N3 (colon* or bowel* or colitis or colitides)**)** or "functional colon*" or colonospasm* or ibs |
|  | 2 | mh "**Irritable Bowel Syndrome+**" |
|  | 3 | **(**(feces or faeces or fecal or faecal or stool*) N3 (enema* or infusion* or transplant* or instill* or tranfus* or donor* or reconstitution* or therap*)**)** or **(**(matter* or microb* or bacteri* or microflora*) N3 (transplant* or transfer*)**) or** bacteriotherap* or FMT |
|  | 4 | mh "Fecal Microbiota Transplantation+" |
|  | 5 | (S1 OR S2) AND (S3 OR S4) |
|  | 6 | S5 AND (MH ("randomized controlled trials" OR "double‐blind studies" OR "single‐blind studies" OR "random assignment" OR "pretest‐posttest design" OR "cluster sample") OR TI (randomised OR randomized) OR AB (random*) OR TI (trial) OR (MH (sample size) AND AB (assigned OR allocated OR control)) OR MH (placebos) OR PT (randomized controlled trial) OR AB (control W5 group) OR MH ("crossover design" OR "comparative studies") OR AB (cluster W3 RCT)) NOT ((MH ("animals+" OR "animal studies") OR TI (animal model*)) NOT MH (human)) |
|  | 7 |  |
|  | 8 |  |
| **Scopus** | 1 | **TITLE-ABS** (**(**(irritable or unstable or nervous or spasm* or spastic* or mucomembran* or mucous*) W/3 (colon* or bowel* or colitis or colitides)**)** or "functional colon*" or colonospasm* or ibs) OR **AUTHKEY** (**(**(irritable or unstable or nervous or spasm* or spastic* or mucomembran* or mucous*) W/3 (colon* or bowel* or colitis or colitides)**)** or "functional colon*" or colonospasm* or ibs) |
|  | 2 | **TITLE-ABS** (**(**(feces or faeces or fecal or faecal or stool*) W/3 (enema* or infusion* or transplant* or instill* or tranfus* or donor* or reconstitution* or therap*)**)** or **(**(matter* or microb* or bacteri* or microflora*) W/3 (transplant* or transfer*)**) or** bacteriotherap* or FMT) OR **AUTHKEY** (**(**(feces or faeces or fecal or faecal or stool*) W/3 (enema* or infusion* or transplant* or instill* or tranfus* or donor* or reconstitution* or therap*)**)** or **(**(matter* or microb* or bacteri* or microflora*) W/3 (transplant* or transfer*)**) or** bacteriotherap* or FMT) |
|  | 3 | ( INDEXTERMS ( "clinical trials" OR "clinical trials as a topic" OR "randomized controlled trial" OR "Randomized Controlled Trials as Topic" OR "controlled clinical trial" OR "Controlled Clinical Trials" OR "random allocation" OR "Double-Blind Method" OR "Single-Blind Method" OR "Cross-Over Studies" OR "Placebos" OR "multicenter study" OR "double blind procedure" OR "single blind procedure" OR "crossover procedure" OR "clinical trial" OR "controlled study" OR "randomization" OR "placebo" ) ) OR ( TITLE-ABS-KEY ( ( "clinical trials" OR "clinical trials as a topic" OR "randomized controlled trial" OR "Randomized Controlled Trials as Topic" OR "controlled clinical trial" OR "Controlled Clinical Trials as Topic" OR "random allocation" OR "randomly allocated" OR "allocated randomly" OR "Double-Blind Method" OR "Single-Blind Method" OR "Cross-Over Studies" OR "Placebos" OR "cross-over trial" OR "single blind" OR "double blind" OR "factorial design" OR "factorial trial" ) ) ) OR ( TITLE ( clinical trial OR trial OR rct* OR random* OR blind* ) ) |
|  | 4 | #1 AND #2 AND #3 |
|  |  |  |
