# Supplementary material for: Clinical efficacy and safety of faecal microbiota transplantation in the treatment of irritable bowel syndrome: a systematic review, meta-analysis and trial sequential analysis
Source: Eur J Med Res. 2024 Sep 18;29:464. doi: 10.1186/s40001-024-02046-5 (PMC11409544; doi:10.1186/s40001-024-02046-5)
Supplement: Supplementary file 2 — Supplementary material 2: Table 2. GRADE evidence profile: FMT for patients with IBS [file 40001_2024_2046_MOESM2_ESM.docx]

**Table S2. GRADE evidence profile: FMT for patients with IBS**

**Clinical Response**

| **Certainty assessment** | | | | | | | **№ of patients** | | **Effect** | | **Certainty** | **Importance** |
| --- | --- | --- | --- | --- | --- | --- | --- | --- | --- | --- | --- | --- |
| **№ of studies** | **Study design** | **Risk of bias** | **Inconsistency** | **Indirectness** | **Imprecision** | **Other considerations** | **Clinical response** | **placebo** | **Relative (95% CI)** | **Absolute (95% CI)** |  |  |
| **Clinical response: Overall** | | | | | | | | | | | | |
| 11 | randomised trials | serious^a^ | serious^b^ | not serious | serious^c,d^ | none | 219/345 (63.5%) | 98/244 (40.2%) | **RR 1.44** (0.88 to 2.33) | **177 more per 1,000** (from 48 fewer to 534 more) | ⨁◯◯◯ Very low | IMPORTANT |
| **Clinical response: FMT via oral capsule** | | | | | | | | | | | | |
| 4 | randomised trials | serious^a^ | serious^b^ | not serious | serious^c,d^ | none | 29/68 (42.6%) | 41/70 (58.6%) | **RR 0.74** (0.33 to 1.68) | **152 fewer per 1,000** (from 392 fewer to 398 more) | ⨁◯◯◯ Very low | IMPORTANT |
| **Clinical response: FMT via endoscopy/nasojejunal tube/rectal enema** | | | | | | | | | | | | |
| 7 | randomised trials | serious^a^ | serious^b^ | not serious | serious^c^ | none | 190/277 (68.6%) | 57/174 (32.8%) | **RR 1.91** (1.26 to 2.91) | **298 more per 1,000** (from 85 more to 626 more) | ⨁◯◯◯ Very low | IMPORTANT |
| **Clinical response: Low risk of bias** | | | | | | | | | | | | |
| 2 | randomised trials | not serious | not serious | not serious | serious^c^ | none | 95/118 (80.5%) | 14/63 (22.2%) | **RR 3.53** (2.21 to 5.64) | **562 more per 1,000** (from 269 more to 1,000 more) | ⨁⨁⨁◯ Moderate | IMPORTANT |
| **Clinical response: Some concerns/high risk of bias** | | | | | | | | | | | | |
| 8 | randomised trials | serious^a^ | serious^b^ | not serious | serious^c,d^ | none | 116/217 (53.5%) | 82/171 (48.0%) | **RR 1.07** (0.69 to 1.65) | **34 more per 1,000** (from 149 fewer to 312 more) | ⨁◯◯◯ Very low | IMPORTANT |

**CI:** confidence interval; **RR:** risk ratio

**Change in IBS-SSS**

| **Certainty assessment** | | | | | | | **№ of patients** | | **Effect** | | **Certainty** | **Importance** |
| --- | --- | --- | --- | --- | --- | --- | --- | --- | --- | --- | --- | --- |
| **№ of studies** | **Study design** | **Risk of bias** | **Inconsistency** | **Indirectness** | **Imprecision** | **Other considerations** | **IBS-SSS** | **placebo** | **Relative (95% CI)** | **Absolute (95% CI)** |  |  |
| **Change in IBS-SSS: Overall** | | | | | | | | | | | | |
| 11 | randomised trials | serious^a^ | serious^b^ | not serious | serious^c,d^ | none | 304 | 235 | - | SMD **0.31 lower** (0.72 lower to 0.09 higher) | ⨁◯◯◯ Very low | IMPORTANT |
| **Change in IBS-SSS: FMT via oral capsule** | | | | | | | | | | | | |
| 4 | randomised trials | serious^a^ | serious^b^ | not serious | serious^c,d^ | none | 64 | 69 | - | SMD **0.16 lower** (1.22 lower to 0.9 higher) | ⨁◯◯◯ Very low | IMPORTANT |
| **Change in IBS-SSS: FMT via endoscopy/nasojejunal tube/rectal enema** | | | | | | | | | | | | |
| 7 | randomised trials | serious^a^ | not serious | not serious | serious^c^ | none | 240 | 166 | - | SMD **0.43 lower** (0.73 lower to 0.13 lower) | ⨁⨁◯◯ Low | IMPORTANT |
| **Change in IBS-SSS: Low risk of bias** | | | | | | | | | | | | |
| 2 | randomised trials | not serious | not serious | not serious | serious^c^ | none | 117 | 63 | - | SMD **0.66 lower** (0.99 lower to 0.33 lower) | ⨁⨁⨁◯ Moderate | IMPORTANT |
| **Change in IBS-SSS: Some concerns/high risk of bias** | | | | | | | | | | | | |
| 8 | randomised trials | serious^a^ | serious^b^ | not serious | serious^c,d^ | none | 177 | 162 | - | SMD **0.09 lower** (0.56 lower to 0.38 higher) | ⨁◯◯◯ Very low | IMPORTANT |

**CI:** confidence interval; **SMD:** standardised mean difference

**Change in IBS-QOL**

| **Certainty assessment** | | | | | | | **№ of patients** | | **Effect** | | **Certainty** | **Importance** |
| --- | --- | --- | --- | --- | --- | --- | --- | --- | --- | --- | --- | --- |
| **№ of studies** | **Study design** | **Risk of bias** | **Inconsistency** | **Indirectness** | **Imprecision** | **Other considerations** | **IBS-QOL** | **placebo** | **Relative (95% CI)** | **Absolute (95% CI)** |  |  |
| **Change in IBS-QOL: Overall** | | | | | | | | | | | | |
| 8 | randomised trials | serious^a^ | serious^b^ | not serious | serious^c,d^ | none | 247 | 177 | - | SMD **0.3 higher** (0.09 lower to 0.69 higher) | ⨁◯◯◯ Very low | IMPORTANT |
| **Change in IBS-QOL: FMT via Oral capsule** | | | | | | | | | | | | |
| 4 | randomised trials | serious^a^ | serious^b^ | not serious | serious^c,d^ | none | 64 | 69 | - | SMD **0.03 higher** (0.78 lower to 0.85 higher) | ⨁◯◯◯ Very low | IMPORTANT |
| **Change in IBS-QOL: FMT via endoscopy/nasojejunal tube/rectal enema** | | | | | | | | | | | | |
| 4 | randomised trials | serious^a^ | not serious | not serious | serious^c^ | none | 183 | 108 | - | SMD **0.53 higher** (0.2 higher to 0.86 higher) | ⨁⨁◯◯ Low | IMPORTANT |
| **Change in IBS-QOL: Low risk of bias** | | | | | | | | | | | | |
| 2 | randomised trials | not serious | not serious | not serious | serious^c^ | none | 117 | 63 | - | SMD **0.77 higher** (0.45 higher to 1.09 higher) | ⨁⨁⨁◯ Moderate | IMPORTANT |
| **Change in IBS-QOL: Some concerns/high risk of bias** | | | | | | | | | | | | |
| 6 | randomised trials | serious^a^ | serious^b^ | not serious | serious^c,d^ | none | 130 | 114 | - | SMD **0.16 higher** (0.31 lower to 0.64 higher) | ⨁◯◯◯ Very low | IMPORTANT |

**CI:** confidence interval; **SMD:** standardised mean difference

#### Explanations

a. ≧ 1 enrolled randomized control trial with a high overall risk of bias.

b. I2＞60%.

c. The sample size was insufficient.

d. CI crossed the threshold.
