# Supplementary figures and images for: Clinical efficacy and safety of faecal microbiota transplantation in the treatment of irritable bowel syndrome: a systematic review, meta-analysis and trial sequential analysis
Source: Eur J Med Res. 2024 Sep 18;29:464. doi: 10.1186/s40001-024-02046-5 (PMC11409544; doi:10.1186/s40001-024-02046-5)

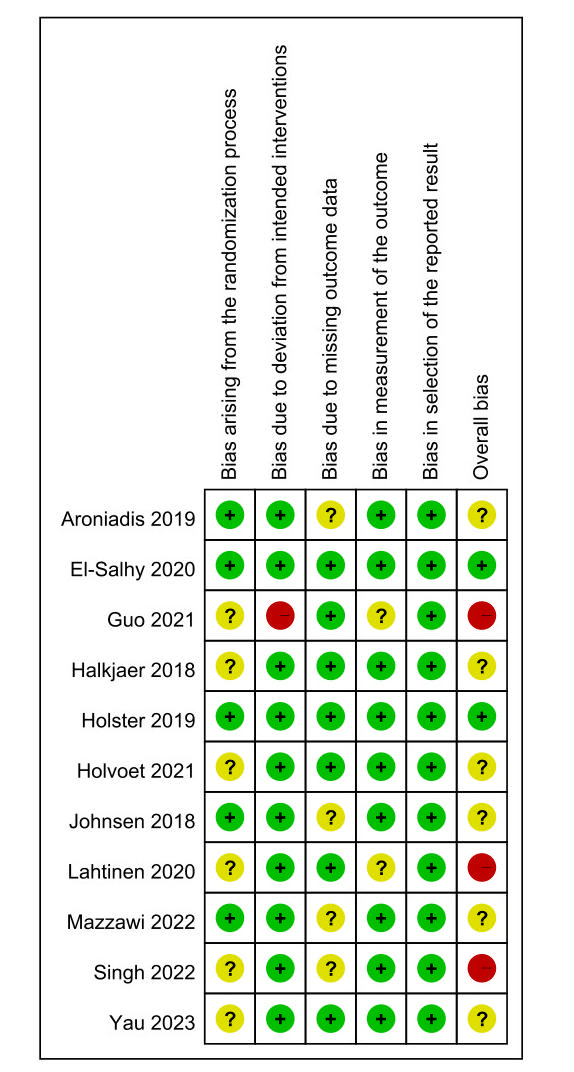

Supplement: Supplementary file 3 — Supplementary material 3: Figure 1. RoB summary [file 40001_2024_2046_MOESM3_ESM.tif]
